# Beyond the Colours of Hydrogen: Opportunities for Process Systems Engineering in Hydrogen Economy

**DOI:** 10.1007/s41660-023-00324-z

**Published:** 2023-03-15

**Authors:** Yick Eu Chew, Xin Hui Cheng, Adrian Chun Minh Loy, Bing Shen How, Viknesh Andiappan

**Affiliations:** 1 Department of Chemical and Environmental Engineering, University of Nottingham Malaysia, 43500 Semenyih, Selangor, Malaysia; 2 School of Engineering and Physical Sciences, Heriot-Watt University Malaysia, 1, Jalan Venna P5/2, Precinct 5, 62200 Putrajaya, Wilayah Persekutuan, Malaysia; 3 Department of Chemical and Biological Engineering, Monash University, Melbourne, VIC 3800 Australia; 4 Biomass-Waste-to-Wealth Special Interest Group, Research Centre for Sustainable Technologies, Faculty of Engineering, Computing and Science, Swinburne University of Technology Sarawak, Jalan Simpang Tiga, 93350 Kuching, Sarawak Malaysia

**Keywords:** Hydrogen economy, Hydrogen value chains, Process systems engineering, Energy trilemma

## Abstract

In the midst of a climate crisis, alternative and low-carbon energy resources must be put to scale in order to achieve carbon emission reductions in the coming decades. In this respect, hydrogen has gained attention as an alternative energy carrier. Hydrogen can be produced from methods that are commonly classified by a range of colours. However, each hydrogen source has its own challenges in terms of energy security, energy equity, and environmental sustainability. This perspective offers insights about the critical role that Process Systems Engineering (PSE) will play in addressing these key challenges. We also present suggestions on possible future PSE studies in the area of the hydrogen economy.

## Introduction

Fossil fuels are the primary energy sources used to meet energy demands. Unfortunately, these energy sources emit large amounts of carbon each year, causing catastrophic environmental impacts. The alarming rise in carbon emissions has pushed policymakers and researchers to look for ways of decarbonising the energy sector. Experts have indicated that carbon emissions must achieve net-zero levels by 2055 (IEA 2021). To do this, cleaner sources of energy must be implemented to replace fossil fuels soon. Hydrogen is a promising alternative energy carrier that produces no emissions when utilised to meet energy needs. Hydrogen can be produced from many sources. However, hydrogen value chains are yet to be established on a large scale. This is because there is a lack of sustainable hydrogen production, storage, transportation, and distribution strategies. Such an issue requires comprehensive optimisation and decision-making models to determine optimal ways to deploy sustainable hydrogen processes in the future. In light of today’s global energy challenges, it is important for decision-makers to factor dimensions such as energy security, energy equity, and environmental sustainability into decision-making. These three dimensions make up the energy trilemma index proposed by the World Energy Council in 2010 (World Energy Council, n.d.). Energy security refers to the ability to meet present and future demands in a reliable and resilient manner. Energy equity is the ability to provide equitable access to affordable energy resources for consumption. Lastly, environmental sustainability focuses on the capacity to mitigate and avoid climate change impacts. These dimensions are multifaceted and require careful consideration when making decisions related to hydrogen value chains. This is where process systems engineering (PSE) can play an important role.

In this perspective, we look at the different classifications of the hydrogen colour spectrum and the challenges brought forth from the lens of the energy trilemma. Following this, this perspective offers a unique take on how PSE can play a critical role to address the energy trilemma dimensions in hydrogen value chains.

## Classifications of Hydrogen

**Fig. 1:**
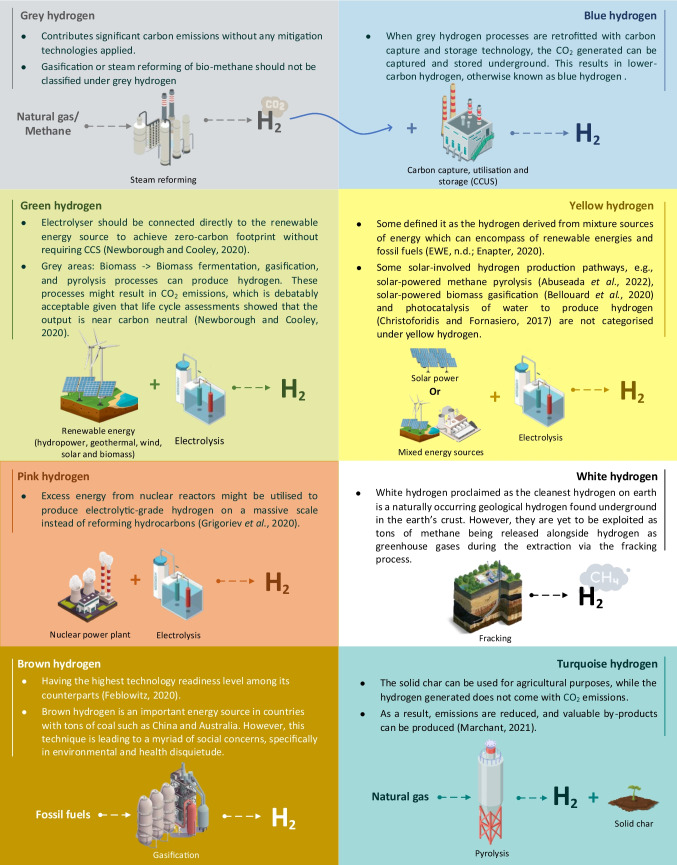
Summary of classifications of hydrogen

Hydrogen is typically classified based on its source. In literature, we may come across a range of categories. Figure 1 offers an overview of the popular categories found in the literature. Alongside this, Table 1 describes and highlights distinctions for each of these classifications.

**Table 1:** Description of each classification

Classification	Description
Grey	• A specific type of fossil-derived hydrogen which are derived from natural gas or methane through steam reforming process (Hulst 2019) • Contributes significant carbon emissions without any mitigation technologies applied • Gasification or steam reforming of bio-methane should not be classified under grey hydrogen
Blue	• The by-product of grey hydrogen is CO_2_. In other words, grey hydrogen processes generate CO_2_ emissions • When grey hydrogen processes (i.e. produced from fossil fuels such as natural gas and coal) are retrofitted with carbon capture and storage technology, the CO_2_ generated can be captured and stored underground (Ajanovic et al. 2022). This results in lower-carbon hydrogen, otherwise known as blue hydrogen (Bauer et al. 2022)
Yellow	• A specific type of green hydrogen which are derived from solar-powered electrolysis (Petrofac, n.d.; Marchant 2021) • Some defined it as the hydrogen derived from mixture sources of energy which can encompass renewable energies and fossil fuels (EWE, n.d.; Enapter 2020) • Some solar-involved hydrogen production pathways, e.g. solar-powered methane pyrolysis (Abuseada et al. 2022), solar-powered biomass gasification (Bellouard et al*.*, 2020), and photocatalysis of water to produce hydrogen (Christoforidis and Fornasiero 2017) are not categorised under yellow hydrogen
Turquoise	• In turquoise hydrogen, pyrolysis is used to crack natural gas into hydrogen and solid char. The solid char can be used for agricultural purposes, while the hydrogen generated does not come with CO_2_ emissions • As a result, emissions are reduced, and valuable by-products can be produced (Marchant 2021)
Green	• Green hydrogen or referred as renewable hydrogen is produced through electrolysis using renewable energy sources (Agaton et al. 2022). These sources included hydropower, geothermal, wind, solar, and biomass • Electrolyser should be connected directly to the renewable energy source to achieve net-zero carbon footprint without requiring CCS (Newborough and Cooley 2020) • Grey areas: biomass fermentation, gasification, and pyrolysis processes can produce hydrogen. It is arguably to be considered under this category since it utilised renewable sources (i.e. biomass) but without the need (not necessarily) for electrolysis. These processes might result in CO_2_ emissions, which is debatably acceptable given that life cycle assessments showed that the output is near carbon neutral (Newborough and Cooley 2020)
Pink	• Pink hydrogen is produced via water electrolysis that is powered by energy generated from a nuclear power station • Excess energy from nuclear reactors might be utilised to produce electrolytic-grade hydrogen on a massive scale instead of reforming hydrocarbons (Grigoriev et al. 2020)
White	• White hydrogen proclaimed as the cleanest hydrogen on earth is a naturally occurring geological hydrogen found underground in the earth’s crust. It is estimated that a total annual flow of white hydrogen (23 Tg/year) is available worldwide and is untapped (Zgonnik 2020) • The reason for untapped white hydrogen is the large amounts of methane that would be released as greenhouse gases alongside hydrogen during the extraction via the fracking process • In order to utilise this technique in the future, well-established methane capture techniques must be integrated into the system • The “white hydrogen” myth states that “white hydrogen is the hydrogen obtained from by-products of industrial processes”, which is a delusion that needs to be clarified
Brown	• Brown, or also known as black hydrogen, is created through the reforming of fossil fuels. The extraction of brown hydrogen is having the highest technology readiness level among its counterparts (Feblowitz 2020) • Brown hydrogen is an important energy source in countries with tons of coal such as China and Australia. However, this technique is leading to a myriad of social concerns, specifically in environmental and health disquietude

The diverse sources of hydrogen discussed in Table 1 indicate significant potential for an alternative source of energy. However, hydrogen value chains need to address several key challenges before global scale-up. This is discussed in the following section.

## Challenges for Hydrogen Economy

The World Energy Council conceived the idea of the energy trilemma index in 2010. The index covers three crucial dimensions that could determine the frontier of energy. These dimensions include energy security, energy equity, and environmental sustainability. With these dimensions in mind, we take a unique look at the challenges of hydrogen value chains via the lenses of the energy trilemma. Table 2 shows a summary of the challenges faced in each hydrogen source, from the energy trilemma perspective.

**Table 2:** Challenges in each hydrogen source from energy trilemma perspective

Classification	Challenges
Grey	Security: Available gas reserves may only be able to sustain for not more than 50 years (BP 2022) Equity: May not retain low prices as gas prices are volatile given market forces Sustainability: Accounts for high carbon intensity (Ewing et al*.*, 2020; IEA 2020)
Blue	Security: CCS at present is energy-intensive, costly, and not 100% efficient (van Renssen 2020) Equity: Price may be higher with the addition of CCS technology (Hieminga and Tillier 2021) Sustainability: May not be environmental as it depends on fossil fuel as the primary feedstock (van Renssen 2020)
Yellow	Security: Intermittent availability of energy source and location dependent Equity: Price inflation of various metals (Farmer 2021), low efficiency, and intermittent nature may cause prices to increase Sustainability: High water consumption (Bracken et al. 2015; Frisvold and Marquez 2013), impact from mining activities for solar panel materials (e.g. silica, silver, and aluminium) and generation of waste materials from solar panels (Walzberg et al. 2021)
Turquoise	Security: Current knowledge on its scale-up is still lacking as mostly are at the lab-scale, where pure methane is used as the feedstock Equity: Dependent on volatile gas prices and low technology readiness may jack up the cost of hydrogen even further (McFarland 2022) Sustainability: Generates solid carbon (i.e. biochar) as a by-product that would be considered as waste and if the biochar can be utilized in agriculture or catalyst development, the whole system would give negative carbon emissions
Green	Security: The operational patterns and scale-up of renewable energy sources are the key bottlenecks for the production of green hydrogen Equity: High price of renewable energy to compensate for intermittency issues Sustainability: End-life management of materials used in generating renewable energy will be an issue (Mishnaevsky 2021)
Pink	Security: Low overall thermal efficiency (Crosbie and Chapin 2003) Equity: A high carbon tax is necessary to make pink hydrogen feasible (Motazedi et al. 2021) Sustainability: The disposal of radioactive wastes without proper handling will contaminate the environment and be dangerous to human health
White	Security: Methane blooming and earthquake disaster might happen after the extraction of hydrogen (Energy observer, n.d.) Equity: Uncertain due to lack of implementation to date Sustainability: Unclear evidence of side effect from the extraction towards the environment (Ortigao 2020)
Brown	Security: Not accessible globally, available in limited locations (Megía et al. 2021) Equity: Not all countries have access to coal, especially those that are facing an energy crisis from the economic fall-out of the COVID-19 pandemic Sustainability: Currently, brown hydrogen production emits about 900 million tonnes of carbon dioxide annually, which is one of the biggest contributors to GHG (Hancock and Ralph 2021)

As shown in Table 2, each hydrogen source faces challenges in terms of security, equity, and sustainability. For instance, grey hydrogen is highly dependent on fossil fuel availability. Fossil fuel such as natural gas is a non-renewable source that has finite availability. Based on the reserve-to-production (R/P) ratio (i.e. the remaining duration that those existing reserves would last if the same production rate is applied), it can be deduced that the available gas reserves may only be able to sustain for not more than 50 years (BP 2022). Meanwhile, the presumption that grey hydrogen will retain low prices is no longer reliable given the market forces (e.g. the implementation of carbon penalty), and the potential volatility of gas prices (e.g. the recent Russia-Ukraine conflict causing a surge in prices) (IEA 2022a). This poses a threat in terms of energy equity. Moreover, the sustainability of grey hydrogen is questionable as it accounts for high carbon intensity, up to 12.1 kgCO_2_/kg hydrogen (Ewing et al. 2020; IEA 2020). This is attributed to the 830 Mt CO_2_ of annual carbon emissions (IEA, 2022b).

Blue hydrogen, on the other hand, faces challenges in carbon capture and storage (CCS). CCS at present is not 100% efficient in removing emissions; therefore, affecting its reliability. CCS technologies are also energy-intensive, which can be very costly (van Renssen 2020). With rising gas prices, the cost of processing natural gas into blue hydrogen will undoubtedly increase. This, in turn, creates an issue regarding the accessibility of blue hydrogen due to its potentially high price. With the addition of CCS technology, the price may rise even further (Hieminga and Tillier 2021). Blue hydrogen may not be as environmentally friendly since it depends on fossil fuel as the primary feedstock (van Renssen 2020).

In yellow hydrogen, the intermittent nature of solar energy (e.g. availability of the solar radiation is not constant throughout the day in a given location) has caused significant concerns in sustainability (Asiaban et al. 2021). Moreover, the daily radiation pattern is anticipated to vary in future climates (Patchali et al. 2020; Wild et al. 2015) which may amplify these challenges to ensure long-term sustainable yellow hydrogen supply. Despite the expectation of a steady drop in solar energy system prices (IRENA 2021), the investment costs required for solar panels or modules are anticipated to increase significantly given to price inflation of various metals, including magnesium, silver, and aluminium (Farmer 2021). Aside from this, energy efficiency of electrolysis is merely between 40 and 60% (Germscheidt et al. 2021), while the low energy efficiency (~ 20% (IRENA 2019)) of solar panels may aggravate this issue further. On the other hand, yellow hydrogen can be used as a media to store electricity from intermittent solar energy (i.e., Power-to-X). However, this intermittent nature makes the investment required for such an operation infeasible (Palys and Daoutidis 2022). Yellow hydrogen may also bring environmental challenges. Solar power can reduce water usage by up to 15% compared to coal and natural gas power plants (Bellini 2019). However, a significant amount of water consumption (Bracken et al. 2015; Frisvold and Marquez 2013) is still expected. Apart from this, mining activities for materials to build solar panels (e.g. silica, silver, and aluminium) may lead to unexpected threats to biodiversity (Sonter et al. 2020). According to Heath et al. (2020), about 80 million tonnes of solar panels will reach their lifespan. These waste materials have to be treated to avoid severe environmental damage (Walzberg et al. 2021).

Turquoise hydrogen reduces the number of complex steps. However, current knowledge on its scale-up is still lacking. To date, most works in this area are at the lab-scale, where pure methane is used as the feedstock. This may put its reliability into question when dealing with large volumes of natural gas (Sánchez-Bastardo et al*.*, 2021). As with blue hydrogen, turquoise hydrogen faces similar challenges related to gas prices. However, the impact may be even more severe as methane pyrolysis is still not ready for commercialisation. This may jack up the cost of hydrogen even further (McFarland 2022). As for sustainability, turquoise hydrogen processes generate solid carbon (i.e., char) as a by-product. Although these processes reduce carbon emissions, its value hinges on how the solid char is used. If there is no uptake in the market, the solid char would be considered waste.

For green hydrogen, the operational patterns and capacity factors of renewable energy sources are the key bottlenecks for the production of green hydrogen. Fortunately, unlike electricity, hydrogen can be scaled up and stored economically (Newborough and Cooley 2020). Although green hydrogen generation with biomass promises zero to negative emissions, it is more difficult to scale up than electrolysis since the amount of indigenous biomass often meets a small portion of the nation’s energy requirement (Newborough and Cooley 2020; Klinge 2021). In addition, given that the freshwater makes up below 1% of the earth’s water, the requirement of about 9 kg of water per kg of hydrogen produced will inevitably add a burden to the water cycle (Beswick et al. 2021). While the proposal of using seawater for electrolysis has a potential in solving this concern (Gao et al. 2022), the need of additional investment for desalination process creates another conundrum to the equity aspect. Similar to yellow hydrogen, the current challenge for green hydrogen is the higher price of renewable power compared to grey and blue hydrogen (Nicita et al. 2020) and also the requirement of significant capital investment to compensate the intermittency issue. Additionally, due to electrolyser expenses and conversion losses, green hydrogen will be more expensive than the electricity used to produce it (Cloete et al. 2021). This needs to be solved by having more renewable energy surplus. In terms of sustainability, the end-life management of materials used in generating renewable energy will be an issue. For instance, wind turbine blades are problematic to recycle as they are made up of various material elements such as glass and carbon fibre-reinforced composites (Mishnaevsky 2021).

Pink hydrogen suffers from low overall thermal efficiency. The efficiency of the electrolysis process is often about 75%, whereas the efficiency of producing electricity is typically around 30%. This indicates that the overall thermal efficiency for the production of pink hydrogen using conventional electrolysis technology is around 25% (Crosbie and Chapin 2003). As the current price of pink hydrogen is expensive, a carbon tax of $ 360/tonne CO_2e_ is necessary to make pink hydrogen which utilises high temperature steam electrolysis economically competitive with grey hydrogen (Motazedi et al. 2021). The sustainability of pink hydrogen is also doubtful. As pink hydrogen is powered by nuclear facilities, the disposal of radioactive wastes without proper handling will contaminate the environment and be dangerous to human health (Verfondern et al*.*, n.d.).

For white hydrogen, studies have hypothesised that hydrogen degassing from mother nature’s core might not be that straightforward, as methane blooming and earthquake disasters might happen after the extraction of hydrogen. Such formidable challenges and uncertainties need to be considered thoroughly before this technology is unveiled to the market (Energy observer, n.d.). Since white hydrogen is still a long way from being implemented, it is unclear how it would impact energy prices. In this respect, white hydrogen remains uncertain in terms of equity. This is because the affordability of the hydrogen degassing technology still remains uncertain, and thus, the cost of the hydrogen obtained still cannot be predicted on an industrial scale. Besides, white hydrogen degassing technology still remains unsustainable, or at least, until more evidence proves that there is a minimal side effect of the extraction towards the environment. Most importantly, the hydrogen yield obtained from this technique solely is insufficient to adequate for the global demand for hydrogen sustainability feasible (Ortigao 2020).

Lastly, brown hydrogen faces issues such as the limited availability of coal reserves globally, which threatens its security (Megía et al. 2021). It is the cheapest hydrogen source compared to other counterparts. Evidently, several multinational companies are well-established in using brown hydrogen (i.e. Kawasaki Heavy Industries, Air Liquide, Mitsubishi Cooperation and Hydrogen Energy Supply Chain Latrobe Valley (HESC 2022; Reuters 2017; National Grid, 2022). According to the BP energy report (2019), the consumption of coal as brown hydrogen feedstock is not falling at an alarming rate and is sufficient for more than one century. On the flip side, not all countries have access to coal, especially those that are facing an energy crisis from the economic fall-out of the COVID-19 pandemic. Currently, brown hydrogen production emits about 900 million tonnes of carbon dioxide annually, which is one of the biggest contributors to GHG (Hancock and Ralph 2021). Despite this, brown hydrogen is not expected to be phased out in the coming decades, mainly due to a lack of suitable resources that could substitute it completely.

Aside from the unique challenges of each hydrogen source, there is a need to establish dedicated infrastructure for logistics in hydrogen value chains. New infrastructure may incur intensive costs which will be passed over to the consumer to bear. This would make hydrogen supplied energy unaffordable and affect energy equity. A possible solution for this is to blend hydrogen with natural gas as an interim start-up measure before venturing completely into new hydrogen infrastructure (Razi and Dincer 2022). However, this is subjected to location, pipeline availability, further negotiations with relevant stakeholders and the trade-off between cost and sustainability. On the other hand, the highly flammable nature of hydrogen is perceived by society as high safety risk. There are pre-set perceptions that hydrogen storage and transportation is unsafe. In addition, storage of hydrogen at present is very costly and has low storage capacity (Eljack and Kazi 2021). This might cast doubts on the sustainability of hydrogen.

## Role of PSE as an Enabler for Hydrogen Economy

The challenges described in the previous section indicate that there is still a lot of work to be done in hydrogen value chains. As mentioned in the introduction, comprehensive and holistic decision-making is important for the realisation of hydrogen utilisation. This is where Process Systems Engineering (PSE) can play a vital role. PSE has a rich history of developing decision-making tools for process synthesis. Process synthesis is defined as “an act of determining the optimal interconnection of processing units as well as the optimal type and design of the units within a process system” (Nishida et al. 1981). The idea of “process synthesis” has many parallels with the hydrogen problem discussed in this perspective. At the present time, we see a rise in new hydrogen production technologies and a steady drop in the respective investment costs due to improving learning rates. Therefore, it is important to have suitable decision-making tools to determine the optimal hydrogen production, storage, transportation, and distribution systems based on cost, safety, environmental impact, etc.

**Fig. 2:**
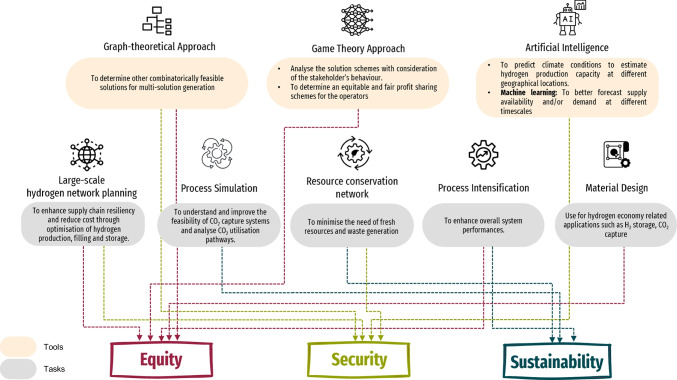
Mapping of PSE tools to targeted challenges in energy trilemma

The field of PSE is known for its contributions to process modelling, optimisation, simulation, and planning systems (Stephanopoulos and Reklaitis 2011). Recently, new developments in PSE cover areas like data analytics, artificial intelligence, and machine learning (Pistikopoulos et al. 2021; Lee et al. 2018). Nevertheless, PSE will play a vital role in addressing challenges posed by the energy trilemma. Figure 2 and Table 3 map out how future PSE tools can address targeted challenges within the energy trilemma.

**Table 3:** PSE enablers for targeted challenges within the energy trilemma

Enablers	Targeted challenges
Large-scale hydrogen network planning (i.e. nationwide (Lim et al. 2021) and region-wide hydrogen supply network synthesis (Hong et al. 2021) with consideration of diverse hydrogen production feedstock to enhance the resiliency of the supply chain. The production, filling, storage, terminal process, and end use of hydrogen must also be optimised to enhance the overall techno-economic feasibility of the hydrogen supply chain (Agrawal et al. 2005; Hong et al. 2022)	Security, equity
Process simulation studies play an important role in understanding and improving the feasibility of CO_2_ capture systems to decarbonise hydrogen produced from fossil fuels. This can also be extended to analyse pathways for CO_2_ utilisation. Simulation studies can also be linked to the development of digital twins. Digital twins may serve as a virtual representation of hydrogen production plants and will involve key PSE knowledge such as thermodynamics, process dynamics, and separations). In addition, PSE has a dedicated domain focusing on first principle modelling studies, which can be integrated with material studies. Material studies on CO_2_ capture absorbents are important in informing first principle modelling studies. This is because first principle modelling studies are used to model performance variables and subsequently optimise them for the feasibility of CO_2_ capture systems	Sustainability
Resource conservation network for intra-plant (Chen et al. 2018) and inter-plant (Lou et al. 2019) hydrogen network synthesis to minimise the need of fresh resources and waste generation. Such intervention can be extended to the upstream (i.e. power generation), where the produced wastes (e.g. the photovoltaic waste from yellow hydrogen production (Walzberg et al. 2021)) can be treated or re-purposed and reused in the value chain. These integrations can be evaluated using established process integration and pinch analysis tools (Klemeš and Kravanja 2013)	Security, sustainability
Multi-solution generation using graph-theoretical approach (Friedler et al. 2019) to determine other combinatorially feasible solutions. Note that the identification of sub-optimal solutions is vital as the optimal solution found is not guaranteed practical in real life (Lakner et al. 2022)	Security, equity
Analysing the solution schemes using a game theory-based approach (Affery et al. 2021; Almutairi et al. 2022) that consider the stakeholder’s behaviour to aid decision-making at the policy refurbishment level; while blockchain technology can be integrated into the system to enable secured, transparent, and reliable information across each stakeholder (Loy et al. 2022)	Equity
Predicting climate conditions (e.g. solar radiation (Angela et al. 2011) and wind speed (Fogno Fotso et al. 2022)) using artificial intelligence tools (e.g. artificial neural network (ANN)) to better estimate the hydrogen production capacity based on specific geographical locations. Machine learning tools can be used for forecasting supply availability and/or demand at different timescales. These can serve as parameters that can then be used in the PSE models mentioned above prior to optimisation and planning. Moreover, artificial intelligence tools can be used to predict material properties more accurately for hydrogen storage and carbon capture. This will improve efficiency of these processes and subsequently lower the cost of hydrogen	Security, equity
Applying process intensification (e.g. membrane reactor for steam methane reforming (Li et al. 2022)) to enhance the overall performances (i.e. energy efficiency, production rate, operating and capital costs) (Abdulrahman et al. 2021)	Equity, sustainability
Cooperative game theory approaches are useful in determining equitable and fair profit sharing schemes for operators in hydrogen supply chains. Thus, in turn, will avoid unregulated pricing and translate to more affordable energy pricing for consumers using hydrogen energy	Equity

## Concluding Remarks

As a whole, it is evident that Process Systems Engineering (PSE) will play an important role in addressing key challenges related to the hydrogen economy. Energy security, energy equity, and environmental sustainability issues may pose a stumbling block at present. With well-developed PSE tools in the future, the true potential of the hydrogen economy can be assessed objectively. PSE tools and their extensions are important to inform the debate on hydrogen economy deployment, specifically on which hydrogen resource should be used, how much, and where should they be sourced from, and the overall impact on carbon emissions reduction targets.

## Data Availability

All data generated or analysed during this study are included in this published article (and its supplementary information files).
